# What are the fundamental ways that psychiatric services should engage with carers and family?

**DOI:** 10.1192/j.eurpsy.2023.411

**Published:** 2023-07-19

**Authors:** D. Maybery

**Affiliations:** Monash University, Warragul, Australia

## Abstract

**Introduction:**

When mental health service providers, service users and their carers/family are successfully integrated, widespread benefits will flow to all stakeholders. However, mental health services do not commonly engage with carers or family.

**Objectives:**

This presentation describes (a) an extensive review of the literature and (b) empirical research with carers and family about what they received and wanted from engagement with mental health services.

**Methods:**

A mixed method online survey asked 134 family members and carers what they received and what they wanted from mental health services. Participants also quantified the importance of seven hypothesised core practices on a 0-100 point likert scale.

**Results:**

Almost 250 verbatim responses were deductively matched against hypothesised engagement practices from the literature, with additional unaligned responses inductively categorised. The findings triangulate with multiple diverse literatures to confirm seven fundamental engagement practices that carers and family want from health services. Conceptually, these practices are represented by two broad overarching practice themes of (i) meeting the needs of the family member and (ii) addressing the needs of the service user.

**Conclusions:**

Policy, clinical practice, training and future research might encompass these core practices along with consideration of the intertwined relationship of family, carers and the service user suggested by the two broader concepts.

**Disclosure of Interest:**

None Declared

